# Saudi patients outcomes after surgical treatment of adolescent idiopathic scoliosis

**DOI:** 10.1186/s13018-023-03925-z

**Published:** 2023-06-23

**Authors:** Abdulmajeed Alzakri, Faisal AlMuhid, Nasser Almousa, Muaath Aljehani, Hashem Alhalabi

**Affiliations:** 1 Department of Orthopedics, College of Medicine, King Saud University, Riyadh, Saudi Arabia; 2 King Saud University Medical City, King Saud University, Riyadh, Saudi Arabia

**Keywords:** Adolescent idiopathic scoliosis, Major Cobb angle, Surgery, Quality-of-life and, Satisfaction

## Abstract

**Background:**

In order to improve post-operative patient’s quality-of-life, attention must be paid to the identification of factors that play a role in patient’s satisfaction with surgical treatment. Patient satisfaction with the outcomes of scoliosis surgery has not been addressed so comprehensively in Saudi literature, as well as the effect of patient satisfaction on the different domains of the SRS-22 questionnaire and radiographic parameters is yet to be reported locally. The aim of this study is to explore such findings especially in our population.

**Methods:**

A retrospective cohort study was conducted at two of the largest centers of spine surgery in the Kingdom of Saudi Arabia. A total of 316 eligible patients were selected via consecutive sampling technique. Data were collected from eligible patients who fit our inclusion criteria, which includes patients with adolescent idiopathic scoliosis aged from 10 to 21 years. Student t test, Pearson’s and Spearman’s correlation coefficients statistical tests were used.

**Results:**

There were 283 (89.6%) females and 33 (10.4%) male patients with a mean age of 15.09 (± 2.27 SD) years. All the domains of SRS-22 showed significantly higher scores 2-year postoperatively, when compared with preoperative values (*P* < 0.001). The change in all SRS-22 domains correlated positively and significantly with the 2-year postoperative satisfaction using Pearson’s correlation coefficient (*P* < 0.05); the total score showed the highest correlation followed by the self-image domain. The major Cobb angle correction percentage correlated significantly (*P* < 0.05) solely with the change in pain domain.

**Conclusion:**

Self-image correlated highly and significantly with patient satisfaction postoperatively. It also had the biggest influence SRS-22 scores postoperatively in conjunction with satisfaction scores. That is an indication of the role it plays in patient satisfaction and quality-of-life postoperatively, which may influence the surgical decision making.

**Supplementary Information:**

The online version contains supplementary material available at 10.1186/s13018-023-03925-z.

## Introduction

The Scoliosis Research Society (SRS) defines adolescent idiopathic scoliosis (AIS) as a spinal deformity with a coronal Cobb angle greater than 10 degrees [1]. With a prevalence estimate of 2–3%, AIS is the most common cause of three-dimensional deformities of the spine [2, 3]. Although it is mostly asymptomatic and non-life-threatening, the deformities stemming from AIS can have a major impact on a patient’s quality-of-life, leading to concerns about physical appearance, pain, and limitation of function [1, 2, 4]. The main goal of surgical treatment of AIS is the correction of the deformity, alleviating future morbidity related to the worsening of the deformity. Because of the benignity of presentation, operative treatment of AIS is expected to yield excellent results, barring significant complications. However, like any other surgery, it carries its share of possible complications and the risk of poor outcomes [2, 4–7].

Historically, the postoperative result of adolescent idiopathic scoliosis has always been focused on the radiological parameters and the percent of curve correction that has been obtained. However, these parameters do not necessarily correlate with patient satisfaction and overall improvement in quality-of-life [4]. Despite satisfactory clinical, surgical and radiological outcomes, patient self-evaluation and quality-of-life may be poor. Several factors could be involved, including age, ethnicity, sociocultural issues, body mass index (BMI), and gender [8]. Taking into consideration the magnitude of effect these variables have on a patient’s postoperative quality-of-life, makes the identification of such variables critical in knowing how to properly manage patients with AIS [3].

Various quality-of-life questionnaires have been developed to aid in this matter. One such questionnaire is the Scoliosis Research Society (SRS)-22 questionnaire. Being the go-to questionnaire for patients with scoliosis, it measures five domains including pain, activity, appearance, mental health, and satisfaction [4]. In order to improve postoperative patient’s quality-of-life, attention must be paid to the identification of factors that play a role in patient’s satisfaction with surgical treatment. Thus far, patient satisfaction with the outcomes of AIS surgery has not been addressed so comprehensively in Saudi literature, as well as the effect of patient satisfaction on the different domains of the SRS-22 questionnaire and radiographic parameters is yet to be reported locally. [1, 6]. Some studies have found a statistically significant correlation between patient satisfaction and radiographic parameters [5], while others have found that the cosmetic appearance correlated with patient satisfaction [9, 10]. However, as no such study of this magnitude has been conducted here, such inferences cannot be made of the Saudi population. Taking into consideration Saudi Arabia’s distinctive culture, the fact that scoliosis patients present late, and the fact that surgical treatment is mostly rejected by Saudi patients. These considerations make it crucial to identify which factors will affect patient’s quality-of-life in Saudi population in order to aid surgical treatment plan [11].

## Methods

### Hypothesis

The authors of this study postulate that the 2-year postoperative measurements of the SRS-22 domains will improve significantly when compared to their preoperative counterparts. The authors went on to postulate that since the greater part of their sample were females, combined with the fact that cosmetic appearance holds great value among people and makes the researchers hypothesize that it will correlate significantly with the patient's satisfaction.

### Study design

This is a retrospective cohort study conducted with the aim to compare quality-of-life outcomes of spinal correction and fusion surgery with the radiological parameters of scoliosis. This study was reviewed by an institutional review board and given subsequent approval (**IRB Project No. E-23–7596).**

### Study setting

This study was conducted at King Khalid University Hospital and King Faisal Specialist Hospital located in Riyadh, Saudi Arabia.

### Sampling technique and target population

The authors of this study have used consecutive sampling technique in their study to include as many patients as possible, due to the small number of patients who present with AIS and are candidates for spinal correction and fusion surgery. Of the patients who followed for two years, a total of 316 patients filled the SRS-22 questionnaire preoperatively were eligible and agreed to participate in the study.

Our study variables include age, BMI, preoperative Cobb angle, major Cobb angle correction percentage, Lenke curve type, use of pain killers, the presence of spondylolisthesis, the surgical approach, thoracoplasty, traction and the use of a brace.

The quality-of-life has been assessed preoperatively, immediately and, two years postoperatively using SRS-22 health-related quality-of-life questionnaire.

### Data collection

Data were collected from eligible patients who followed for two years, filled the SRS-22 questionnaire preoperatively and fit the study’s inclusion criteria, which includes patients with adolescent idiopathic scoliosis, ages 10 to 21 years. The patients with congenital scoliosis or syndromic scoliosis were excluded.

Data were collected from patients scheduled for spinal fusion surgery for medical purposes and not as a part of the research project. The Scoliosis Research Society (SRS) developed the SRS-22 questionnaire to evaluate the treatment of patients with adolescent idiopathic scoliosis (AIS). The reason why the researchers chose Scoliosis Research Society (SRS) Outcomes Questionnaire, developed by Haher et al., is that it facilitates the assessment of the outcome of surgical treatment of patients using a simple, practical, idiopathic scoliosis-specific instrument, and from the patient’s perspective [10]. It is available as free material at their website in many languages including English, which was used in this study and given to patients preoperatively.

Participants anonymity was assured by assigning each participant a code number for the sole purpose of analysis, and informed consent was taken from all participants prior to their involvement in the study. The data were collected between May of 2017 and June of 2021.

### Statistical analysis

Data were analyzed using SPSS 27.0 version statistical software. Descriptive statistics (mean, standard deviation, frequencies, and percentages) were used to describe the quantitative and categorical variables. Student t test, Pearson’s and Spearman’s correlation coefficients statistical tests were used. A P value of < 0.05 and 95% CI were used to report the statistical significance and precision of results.

## Results

### Demographic and clinical data

As shown in Table 1, the total eligible participants comprised of 316 patients of whom 89.6% were female and 10.4% were male. The mean age was 15.09 years (± 2.27 years), and their mean BMI showed to be within normal range 20.6 (± 3.9). The most prevalent Lenke curve type was Lenke type 1 (49.68%). Pain killers were used by more than two-thirds (68.4%) of the patients, and spondylolisthesis was only found in 19 patients (6%). The major preoperative Cobb angle had a mean of 61.13 degrees (± 14.18 degrees), while the major postoperative Cobb angle mean was 19.63 degrees (± 8.64 degrees). The major Cobb angle correction percentage had a mean of 71.42% (± 15.26%). Less than half of the patients (43.7%) were braced. Intraoperative traction was used in only 2% of patients. The posterior only approach was the main approach used (78.8%).

**Table 1:** Demographic variables and descriptive characteristics of the sample

Variables	Frequency (n)	Percentage (%)
Sex		
Female	283	89.6
Male	33	10.4
Mean age [range (y)]	15.1 [10–21]	–
Mean Major Pre-op Cobb angle	61.1 (± 14.1)	–
Mean Major Post-op Cobb angle	19.63 (± 8.64)	
Mean Major Cobb angle correction (%)	71.4 (± 15.2)	–
Mean BMI	20.6	–
Use of Painkillers		
Yes	216	68.4
No	100	31.6
Lenke classification		
1	157	49.7
2	35	11.1
3	65	20.6
4	11	3.5
5	18	5.7
6	30	9.5
Brace		
No	178	56.3
Yes	138	43.7
Traction		
No	313	98.4
Yes	3	0.9
Thoracoplasty		
No	306	96.8
Yes	10	3.2
Osteotomy		
No	237	75
Yes	79	25

### SRS-22 domains

In Table 2*,* SRS-22 domains preoperatively and 2-year postoperatively are compared using paired sample t test. All SRS-22 domains showed significant improvement postoperatively (*P* < 0.001), but the largest change was in satisfaction (0.99 ± 1.15) and self-image domains (0.97 ± 0.92), respectively, and the least change was in function domain (0.18 ± 0.65).

**Table 2:** Summary of the change in preoperative and the 2-year postoperative SRS-22 domain scores

Domain	Pre-Op (mean ± SD)	2-year post-op (mean ± SD)	Difference between the means (Mean ± SD)	P*
Pain	3.81 ± 0.79	4.17 ± 0.727	0.36 ± 0.87	< 0.001
Mental	3.85 ± 0.67	4.05 ± 0.709	0.2 ± 0.83	< 0.001
Image	3.21 ± 0.738	4.18 ± 0.670	0.97 ± 0.92	< 0.001
Function	4.12 ± 0.596	4.30 ± 0.456	0.18 ± 0.65	< 0.001
Satisfaction	3.45 ± 0.889	4.44 ± 0.758	0.99 ± 1.15	< 0.001
Total	3.72 ± 0.520	4.20 ± 0.500	0.48 ± 0.59	< 0.001

*A *P* value of less than 0.05 is considered significant

### Years postop satisfaction domain correlation with the change in the other domains

The correlation between the 2-year postoperative satisfaction and the change in SRS-22 domains preoperatively and 2-year postoperatively using Pearson’s correlation coefficient is displayed in Table 3. It shows that the change in all domains preoperatively and 2-year postoperatively correlated positively and significantly (*P* < 0.05) with 2-year postoperative satisfaction. The change in total score showed the highest correlation (0.378) followed by self-image domain (0.332).

**Table 3:** Correlation between 2-year postoperative satisfaction domain and the change in SRS-22 domains

Domain	Correlation coefficient*	P**
Pain	0.176	0.002
Mental	0.202	< 0.001
Image	0.332	< 0.001
Function	0.118	0.037
Total	0.378	< 0.001

*The values were calculated using Pearson’s Correlation coefficient

**A *P* value of less than 0.05 is considered significant

### Cobb angle correlation with the change in SRS-22 domains

Major Cobb angle correction percentage is correlated with the change in SRS-22 domains preoperatively and 2-year postoperatively using Spearman’s correlation coefficient in Table 4. It showed positive correlation with the change in all domains, with the function domain having the least correlation (0.019), but correlate significantly only with pain domain (*P* < 0.05) with a correlation coefficient of (0.112).

**Table 4:** Correlation between the change SRS-22 domain scores and major Cobb angle correction percentage

Domain	Correlation coefficient*	P**
Pain	0.112	0.047
Mental	0.022	0.702
Image	0.048	0.399
Function	0.019	0.736
Satisfaction	0.084	0.135
Total	0.087	0.123

*The values were calculated using Spearman’s Correlation Coefficient

**A P value of less than 0.05 is considered significant

## Discussion

The ever-growing emphasis being put on patient-centered care explains the trend toward assessing AIS treatment success by focusing on patient-centered information, in addition to the conventional radiographic measurements [4]. That is why quality-of-life assessment tools have been developed for the purpose of assessing the benefit of treatment in AIS from patient reported parameters. Scoliosis Research Society (SRS)-22 questionnaire being the go-to questionnaire for patients with scoliosis, it measures five domains including pain, function, appearance, mental health, and satisfaction. Any of these domains might hold the key toward optimizing patient care and achieving the best possible outcome for the patient. Using the SRS-22 questionnaire, combined with the radiological and demographic data, we might be able to assess patient quality of life after surgical treatment of AIS, and the factors directly related to the patient’s satisfaction.

Utilizing the paired sample t test, all the 2-year postoperative SRS-22 domains demonstrated high statistically significant improvement when compared with their preoperative counters (*P* < 0.001). As demonstrated by the results, surgical intervention was associated with an improvement in patient-related outcomes when comparing both preoperative and postoperative data sets in all domains of the SRS-22 questionnaire (pain, satisfaction, mental health, self-image, function, and total scores). The greatest change was observed in the satisfaction domain (0.99 ± 1.15), followed directly by the self-image domain (0.97 ± 0.92). This might hint at a possible relationship between satisfaction and self-image. These results clearly demonstrate the benefit of surgery on AIS in regard with the improvement of quality-of-life indices.

By using Pearson’s correlation coefficient, the change in all SRS-22 domains preoperatively and 2-year postoperatively correlated positively and significantly with 2-year postoperative satisfaction domain (*P* < 0.05), this comes in line with most studies in the literature [3, 4, 6, 11, 12]. The change in total score showed the highest correlation (0.378), followed by self-image (0.332), closely followed by the other domains, especially the mental health (0.202) and pain (0.176) domains. Regardless of their order, all the domains correlated significantly with the satisfaction, indicating that they all played a role in patient overall satisfaction with treatment, however, self-image domain had the highest correlation coefficient, followed by mental health and pain domains, respectively. This might be explained by the fact that the majority (89.6%) of the patients our sample were females, and in general, appearance tends to carry more weight in females. A possible explanation of why the mental-health domain had a correlation coefficient of 0.202, closely following the self-image domain, is the impact self-image has on patient’s overall psychological and mental health. Another domain with a high statistical significance with patients' satisfaction was the pain domain, highlighted by a correlation coefficient of 0.176, indicating its significance in patient satisfaction.

Many previous studies did not find any correlation between Cobb’s angle and the domains of the SRS questionnaire, and only a few have found significant correlation [13–15]. However, using Spearman’s correlation coefficient, this study reported that the major Cobb angle correction percentage correlated with the change in SRS-22 domains preoperatively and 2-year postoperatively. The total score and satisfaction domains had a moderate positive correlation with the major Cobb angle correction percentage, with a correlation coefficient of 0.087 and 0.084, respectively. In other words, greater curve correction results in more satisfaction and a higher total SRS-22 score. The self-image and mental-health domains had a mild positive correlation with the major Cobb angle correction percentage, with a correlation coefficient of 0.048 and 0.022, respectively. The only domain to have a positive significant correlation with the major Cobb angle correction percentage was the pain domain with a correlation coefficient of 0.112, meaning that as the curve correction increased, the pain domain improved, and so did patient quality of life. Based on our findings, if pain was a patient’s main concern, they would highly benefit from a greater surgical curve correction.

In line with our study, *Carreon *et al. demonstrated statistically significant improvements in all SRS domain scores from preoperative to 2-year postoperative, with the largest margin being in the satisfaction domain, which coincides with results from this study. However, the same study has shown low to moderate correlations between the change in domain scores and patient satisfaction with treatment. *Carreon *et al. went on to state that possible explanations for such results could be because of the ceiling effect in the satisfaction domain, poor responsiveness of the questionnaire to effectively measure clinically relevant changes in aspects concerning activity, pain and mental health, or a genuine lack of change in these domains after surgical correction of scoliosis in the adolescent population. On the other hand, some studies have shown a substantial relationship between postoperative satisfaction domain and the change in postoperative SRS domain scores compared to their preoperative counterparts [6] [16–19]. *Hisam *et al. carried out a study involving 37 female patients who underwent surgical treatment for AIS. The study has shown a significant positive correlation in patient satisfaction across all SRS domains postoperatively, with self-image being the least correlated [12].

The correlation between radiographic parameters and the SRS domains has been the crux of many studies concerning AIS throughout the years. Some studies have negated the correlation between the two. A study published by *Linda *et al. found no association between postoperative radiological parameters and the different SRS domains [11]. A more recent study by *Carreon *et al. aimed to define the correlation between radiographic parameters and postoperative satisfaction has shown no statistically significant association between the postoperative SRS satisfaction values and the degree of curve correction (r = 0.078, P = 0.062) [6]. A multi-center study involving 265 adolescents who underwent surgical correction for AIS has shown significant correlations between Cobb angle and postoperative function, total, pain and self-image domains, with the latter having the highest correlation (r = − 0.23, *P* < 0.001) [9]. A recent study published by *Herdea *et al. demonstrated that there are statistically significant correlations between the correction rate and improvements in SRS scores (*P* < 0.001), both in terms of the overall score and within each individual domain of the survey. The conclusion drawn from these findings is that achieving a higher correction rate leads to increased values in the SRS score [20]. In another study conducted by *Ng Bobby*, it was further demonstrated that the degree of curve rectification after surgery and the preoperative maximum Cobb angle serve as significant predictors of various outcomes, such as function scores, self-image, and satisfaction with management in AIS patients. This reinforces the notion that both the magnitude of the surgical correction and the initial severity of the spinal curvature are critical factors in shaping a patient's postoperative functional capacities, their perception of their own physical appearance, and their overall satisfaction with the treatment process [21].

### Limitations

As a retrospective analysis of a prospectively maintained database, might risk subjecting this study to sampling or selection bias. However, the sampling technique utilized in this study was simple consecutive sampling, were the authors excluded only a small amount of patients due to the fact that their data were incomplete. The fact that this study had such a large sample size might alleviate some of these concerns. In the grand scheme of things, these simple issues are negligible when compared to the benefit that our study will provide for the Saudi population.

## Conclusion

All preoperative SRS-22 domains correlated both positively and significantly with their 2-year postoperative counterparts, assuring the patient of the obvious benefit of surgery to their quality-of-life. The change in SRS-22 domains correlated positively with major Cobb angle correction percentage. The fact that self-image highly correlated with satisfaction, along with the fact that it correlated with the major Cobb angle correction percentage, is indicative of the role that it plays in patient satisfaction and quality-of-life postoperatively. However, the major Cobb angle correction percentage correlated significantly with only one SRS-22 domain, the pain domain. Indicating that as curve correction increased, the patient might benefit from an improvement of pain.

## Supplementary Information

**Additional file 1:** The complete data of total 316 patients.

## Data Availability

The dataset supporting the conclusions of this article is included within the article (and its Additional file 1).
